# T2T-CHM13 versus hg38: accurate identification of immunoglobulin isotypes from scRNA-seq requires a genome reference matched for ancestry

**DOI:** 10.1093/nargab/lqaf074

**Published:** 2025-06-11

**Authors:** Junli Nie, Julie Tellier, Ilariya Tarasova, Stephen L Nutt, Gordon K Smyth

**Affiliations:** Walter and Eliza Hall Institute of Medical Research, Parkville, Victoria 3052, Australia; Department of Medical Biology, University of Melbourne, Parkville, Victoria 3010, Australia; Walter and Eliza Hall Institute of Medical Research, Parkville, Victoria 3052, Australia; Department of Medical Biology, University of Melbourne, Parkville, Victoria 3010, Australia; Walter and Eliza Hall Institute of Medical Research, Parkville, Victoria 3052, Australia; Department of Medical Biology, University of Melbourne, Parkville, Victoria 3010, Australia; Walter and Eliza Hall Institute of Medical Research, Parkville, Victoria 3052, Australia; Department of Medical Biology, University of Melbourne, Parkville, Victoria 3010, Australia; Walter and Eliza Hall Institute of Medical Research, Parkville, Victoria 3052, Australia; School of Mathematics and Statistics, University of Melbourne, Parkville, Victoria 3010, Australia

## Abstract

Antibody production by B cells is essential for protective immunity. The clonal selection theory posits that each mature B cell has a unique immunoglobulin receptor generated through random gene recombination and, when stimulated to differentiate into an antibody-secreting cell, has the capacity to produce only a single antibody specificity. It follows from this ‘one-cell-one-antibody’ dogma that single-cell RNA-seq profiling of antibody-secreting cells should find that each cell expresses only a single form of each of the immunoglobulin heavy and light chains. However, when using GRCh38 as the genome reference, we found that many antibody-secreting cells appeared to express multiple immunoglobulin isotypes. When the newly published T2T-CHM13 genome was used instead as the genome reference, every antibody-secreting cell was found to express a unique isotype, and read mapping quality was also improved. We show that the superior performance of T2T-CHM13 was due to its European origin matching the genetic background of the query samples. On the other hand, T2T-CHM13 failed to appropriately fit the ‘one-cell-one-antibody’ dogma when applied to data derived from East Asia. Our results show that read assignment to human immunoglobulin isotype genes is very sensitive to the ancestral origin of the genome reference.

## Introduction

The human genome was first published in 2001 [1–3] and has been continually improved over the past 20 years. For the past decade, the standard human reference genome in use by the UCSC Genome Browser, RefSeq, Ensembl, and Gencode has been the GRCh38 genome released by the Genome Reference Consortium in 2013 [4]. Additional patches have been released regularly, most recently GRCh38.p14 in February 2022 (https://www.ncbi.nlm.nih.gov/datasets/genome/-GCF_000001405.40). GRCh38 has been accepted as a high-quality representation of the 92% of the human genome that is euchromatic and accessible for gene transcription and has been the basis of most human genomic analyses over the past decade [3, 4].

Recently, the telomere-to-telomere (T2T) consortium published the T2T-CHM13 assembly, the first essentially complete sequence of a human genome [5]. The T2T-CHM13 assembly provides complete coverage of heterochromatic and repetitive regions of the human genome and has been shown to improve identification of SNPs and structural variations [6]. Nevertheless, the differences between T2T-CHM13 and GRCh38 for protein-coding genes have been little explored. Most annotated genes and exons are covered by both T2T-CHM13 and GRCh38, and it is difficult to rigorously compare the accuracy of the alternative reference genomes for these common regions unless there is some sort of known ground truth against which to assess the comparison. In this article, we compare T2T-CHM13 and GRCh38 for an important gene family, immunoglobulin isotype genes, for which the ‘one-cell-one-antibody’ dogma provides such ground truth.

B cells and immunoglobins generated by B cells form a major arm of the adaptive immune system [7]. Antigens binding to B-cell receptors, with suitable co-stimulatory signals, activate B cells to proliferate and differentiate into antibody-secreting cells (ASCs) [8]. The antibodies produced by ASCs present a high binding affinity to their target antigens, neutralizing infectious threats, regulating the microbiota, or clearing dead cells from the body [9]. Antibodies are encoded by the immunoglobulin genes (IGH, IGK, IGL) [10–12] and are expressed exclusively by the lymphocytes of the B-cell lineage. An antibody molecule consists of two identical heavy chains and two identical light chains. Both heavy and light chains possess an N-terminal variable region and a C-terminal constant region, with the variable region determining antigen specificity. Based on the type of the constant region of the heavy chain, human immunoglobulins are divided into five major isotypes or classes, IgM, IgD, IgG, IgA, and IgE, among which IgG is further divided into IgG1, IgG2, IgG3, and IgG4, and IgA into IgA1 and IgA2 (Supplementary Fig. S1) [13].

An essential step in the formation of a functional humoral immune system is the somatic genomic recombination that integrates two or three of the segments from the immunoglobulin genes to a functional exon encoding the N-terminal variable region for a particular cell [14] (Supplementary Fig. S1). This process initiates in developing B cells in the bone marrow and occurs in an ordered fashion, where the IGH locus undergoes sequential diversity (D) to joining (J) recombination, followed by variable (V) to DJ recombination, to produce a single VDJ exon encoding the N-terminal variable region of a heavy chain. Upon successful VDJ recombination in one chromosome, the further recombination on the second chromosome is inhibited to ensure that the developing B cell only carries a single functional VDJ rearranged IGH, a process termed allelic exclusion [15, 16]. Following successful IGH rearrangement, developing B cells then undergo a similar (V–J) recombination process in either one of the two immunoglobulin light chain loci (IGK and IGL).

Successful VDJ recombination results in the circulation of mature naïve B cells expressing two forms of immunoglobulin, IgM and IgD, generated by alternative splicing of the same VDJ exon to either an IGHM exon or an IGHD exon (Supplementary Fig. S1) [17]. Once activated, B cells have the unique capacity to replace the IgM/IgD constant regions with other isotypes (IgG, IgA, or IgE) in a process called immunoglobulin class switch recombination (CSR). CSR results in the VDJ exon of the IGH being recombined to any one of the downstream exons encoding the constant regions of IgG, IgE, or IgA by enzymatic deletion of all intervening sequences [18] (Supplementary Fig. S1). The sequence deletion physically ensures that only one isotype is expressed per cell. Key features of the system are that the antibody isotypes have distinct functions [19–24] and that CSR is differentially induced by infections [25–27], mRNA vaccines [28], autoimmune diseases [29], and anti-tumour responses [30]. This process thus allows the type of antibody response to be tailored to meet the challenge at hand. At the completion of this stage, B cells undergo differentiation into ASCs, which transcribe large amounts of a shorter isoform of the IGH mRNA resulting in a secreted antibody form of the protein [31].

An important consequence of the VDJ recombination process outlined above is that each B cell carries only a single functional and unique immunoglobulin molecule. This ‘one-cell-one-antibody’ principle was captured in clonal selection theory proposed by Burnet (the 1960 Nobel laureate) [15], and first proven experimentally by Nossal [32], which underlies the modern industrial production of monoclonal antibodies for research and therapy. Together with the process of CSR, this theory implies that only one immunoglobulin isotype should be present within each single antibody-secreting plasma cell. Advances in single-cell RNA sequencing methods now provide the opportunity to validate this classic immunological concept. Here we show that, when using the GRCh38 (referred to as hg38) as the genome reference for four public datasets, the results appear to violate the ‘one-cell-one-antibody’ dogma. On the other hand, we found that the results agree with the ‘one-cell-one-antibody’ theory perfectly if we switch the reference genome from hg38 to the newly published T2T-CHM13 (T2T) genome. Investigating this phenomenon further, we found that the sequence differences reflect the discrepancies between T2T and hg38 in terms of their origins from different human subpopulations. When using an ancestry-matched genome reference, the identification and quantification of immunoglobulin genes are more accurate.

Given the essential roles of the immunoglobulin isotypes in human health, and the importance of mapping the functionality of B cells in providing insights into the immune responses to infection, vaccination, allergy, and in autoimmune conditions, it is necessary to identify them accurately in RNA sequencing data. This study highlights the importance of considering the reference genome in achieving this goal.

## Materials and methods

### 10x Genomics Chromium single-cell datasets

10x-format FASTQ files were downloaded for each of five public single-cell datasets. The primary dataset focuses specifically on bone marrow plasma cells (BMPCs) [33]. Other datasets were derived from intestinal mucosae [34], tonsil [35], bone marrow [36], and intestinal mucosae from Chinese individuals [37].

The BMPC data were generated using Cell Ranger v5.0.0. Metadata are available from GEO accession GSM7181915 and FASTQ files from SRA accession SRX20001597.

Metadata for the intestine mucosae dataset are available from GEO accession GSM3972026, and FASTQ files were downloaded from SRA accession SRX6584269.

Metadata for the tonsil dataset are available from GEO accession GSM5051497, and a BAM file containing sequence reads was downloaded from SRA accession SRX9986858. The BAM file was converted to FASTQ files using the Cell Ranger v7.0.0 utility bamtofastq.

Metadata for the bone marrow dataset are available from GEO accession GSM3396184, and a BAM file was downloaded from SRA accession SRX4720060. The BAM file was converted to FASTQ files using the Cell Ranger v7.0.0 utility bamtofastq.

Metadata for the Chinese intestinal mucosa dataset are available from GEO accessions GSM3433583 and GSM3433584, and FASTQ files were downloaded from SRA accessions SRX4896886 and SRX4896887.

### Cell Ranger reference genomes

To make a reference genome for Cell Ranger, a FASTA file of genomic sequences and a GTF file of gene annotation are required. For GRCh38, the FASTA file was GRCh38.primary_assembly.genome.fa and the backbone GTF file was gencode.v35.annotation.gtf, both downloaded from https://www.gencodegenes.org. For T2T, the FASTA file was chm13v2.0_maskedY.fa and the backbone annotation was UCSC GENCODEv35 CAT/Liftoff v2, downloaded from https://github.com/marbl/CHM13.

rtracklayer [38] was used to read the GTFs and to facilitate subsetting. Genes were matched between genomes by symbol. The analysis was restricted to protein-coding genes to make the results more reproducible between alternative annotations [39]. Any gene not found in both GRCh38 and T2T annotations was also removed.

FASTA and GTF files for the two Chinese genomes were downloaded from https://ftp.ensembl.org/pub/rapid-release/species/Homo_sapiens/GCA_018471515.1/ and https://ftp.ensembl.org/pub/rapid-release/species/Homo_sapiens/GCA_018472605.1. The two genomes correspond to HG00438 and HG00621 in Figure 1h of [40], but are relabelled as Han-438 and Han-621 in this article to emphasize their Han Chinese origins. Gene annotation for the two Han genome references was restricted to protein-coding genes as for hg38 and T2T.

**Figure 1.**
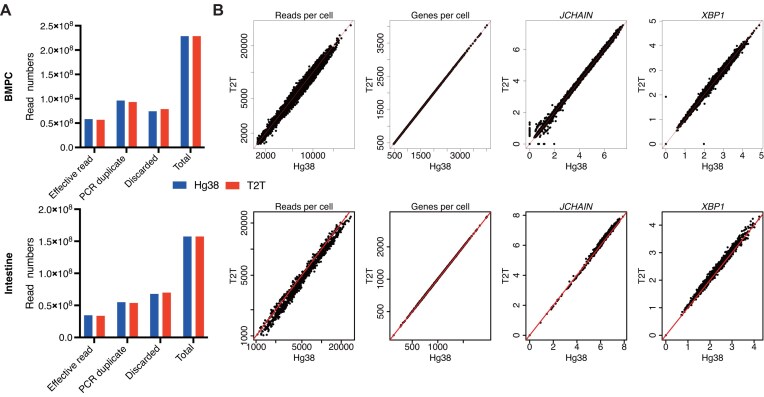
Mapping performance is comparable between hg38 and T2T. (**A**) Classification of reads for the BMPC (top) and intestinal mucosa (bottom) datasets for each reference genome. The bar plots show the total read count for each sample and also the number of effective reads, the number of reads identified as PCR (polymerase chain reaction) duplicates, and the number of reads discarded for other reasons. Only effective reads were used for downstream analyses. (**B**) Reads per cell, genes detected per cell, and expression of two plasma cell marker genes (JCHAIN and XBP1) for the BMPC (top) and intestinal mucosa (bottom) datasets. Here, and subsequently, only *bona fide* ASCs are shown. The hg38 and T2T cell-wise counts and expression values are compared by scatter plots. Expression is represented as the log1p of reads per 10 000 (expression value = log1p(*y*/*L ×*10^4^), where *y* is the read count for the gene and *L* is total read count for the cell). Red lines in scatter plots represent the *x* = *y* line.

Finally, Cell Ranger references were made by cellranger mkref v7.0.0, with the FASTA files and filtered GTF files as inputs.

The custom T2T reference for Fig. 6 was made by editing the GTF file in accordance with Fig. 6A, the remaining process being the same.

### Cell Ranger mapping and quantification

FASTQ files are mapped to GRCh38 and T2T separately. Gene × barcode count matrices were generated by cellranger count v7.0.0.

To generate Figs 1A, 4A, 5A, and 6B, and Supplementary Figs S2, S10, S11D, S12A, S18 (left), andS20A, BAM files generated by cellranger count were used to check the general mapping performance. The xf, GN, AS, and nM tags were extracted from the BAM files by samtools [41]. For each read, GN indicates which gene the read is assigned to, xf indicates the alignment status (effective, PCR duplicate, or discarded), AS gives the mapping score, and nM indicates the edit distance (https://www.10xgenomics.com/support/software/cell-ranger/latest/analysis/outputs/cr-outputs-bam, https://github.com/alexdobin/STAR/blob/master/doc/STARmanual.pdf).

### Quality control and dimension reduction plots

**Figure 2.**
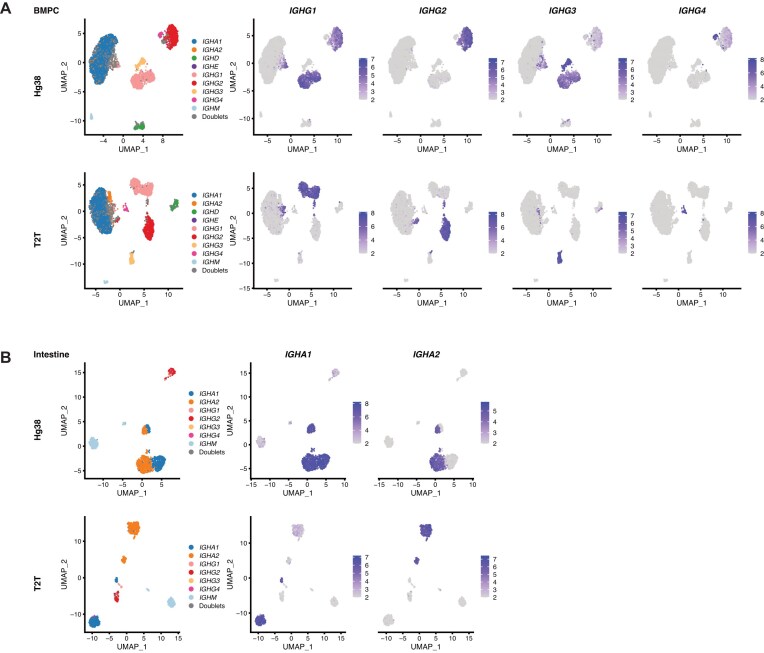
The T2T genome identifies immunoglobulin isotypes more accurately. (**A**) UMAP dimension reduction plots of individual ASCs for the BMPC dataset. The left plots show the isotype assigned to each cell. The isotype assignment was based on the T2T output, in which if an isotype accounts for >85% expression of all isotype gene expression in a single cell, that isotype will be assigned to this single cell. In the right four columns, cells are coloured by expression level of the indicated gene for a particular IgG subclass. Plots are shown for hg38 (top) and T2T (bottom). Here, expression values are represented as the log1p of reads per 10 000 (expression value = log1p(*y*/*L* × 10^4^), where *y* is the read count for the gene and *L* is total read count for the cell). (**B**) UMAP dimension reduction plots of individual ASCs for the intestinal mucosa dataset. The left plots show the isotype assigned to each cell. The isotype assignment was based on the T2T output, in which if an isotype accounts for >85% expression of all isotype gene expression in a single cell, that isotype will be assigned to this single cell. In the right two columns, cells are coloured by expression level of the indicated gene for a particular IgA subclass. Plots are shown for hg38 (top) and T2T (bottom). Here, expression values are represented the same as in panel (A).

**Figure 3.**
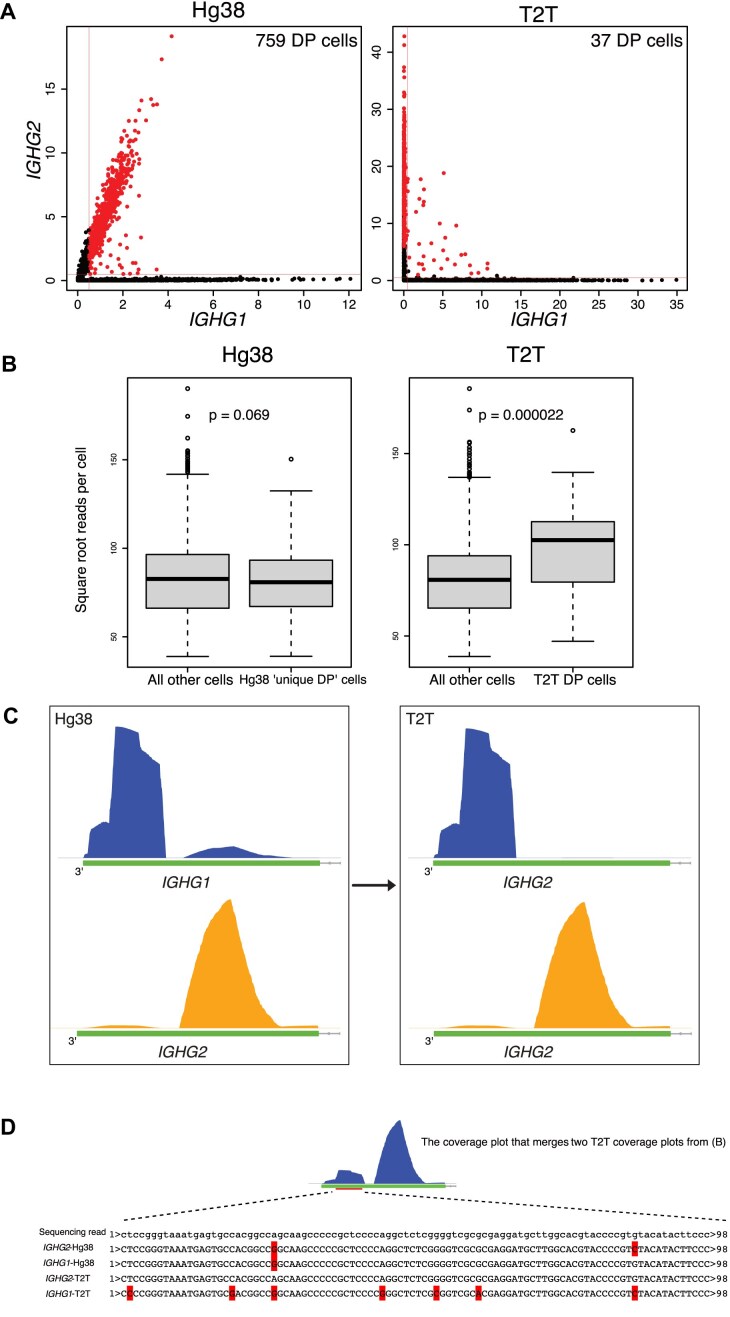
Separating IGHG1^+^ and IGHG2^+^ plasma cells. (**A**) Scatter plots correlating IGHG1 and IGHG2 expression for BMPCs. Left plot for hg38 and right plot for T2T. The IGHG1^+^IGHG3^+^ cells of the hg38 output are highlighted in both plots. The horizontal and vertical red lines represent the thresholds for positive cells of an isotype gene. Here, the thresholds are 0.5% for both IGHG1 and IGHG2. The numbers of double-positive (DP) cells for both outputs are also shown. (**B**) Test whether the DP cells in panel (A) are doublets. We consider (i) 37 DP cells from the T2T alignment, (ii) 759 DP cells from the hg38 alignment minus the T2T DP cells, and (iii) all other 5975 cells. The right box plot shows that the T2T DP cells have significantly larger library sizes than the non-DP cells (two-sided Wilcoxon test, *P* = .000022), confirming that they are likely to be doublets. The left box plot shows the cells that are DP only in the hg38 alignment have library sizes that are similar to or lower than those of the non-DP cells (*P* = .069), confirming that they should be considered as normal cells. (**C**) Coverage plots for reads from the highlighted cells in panel (A). Top left plot shows reads mapped to IGHG1 using hg38 and bottom left shows reads mapped to IGHG2 using hg38. Only the CH3-CHS exon of the genes is shown. The plots on the right show coverage plots for the same reads but using T2T. The top right plot shows that all the reads mapped to IGHG1 using hg38 map instead to IGHG2 using T2T. The bottom right plot shows that all reads mapped to IGHG2 using hg38 map also map to IGHG2 using T2T. (**D**) Alignment results for one illustrative sequence read incorrectly assigned to IGHG1 by hg38 in panel (B). The full sequence of the read is shown together with the best matches to the IGHG2-hg38, IGHG1-hg38, IGHG2-T2T, and IGHG1-T2T genome regions. Mismatched bases are marked in red. The read maps perfectly to IGHG2-T2T and, using T2T, the assignment to IGHG2 is unambiguous. Using hg38, however, the result is reversed with fewer mismatches for IGHG1-hg38 than for IGHG2-hg38. The coverage plot merges the right two plots from panel (B) on the same scale, i.e. shows IGHG2-T2T coverage for all reads mapped by hg38 to either IGHG1 or IGHG2. The aligned position of the illustrative read is shown by a red bar.

**Figure 4.**
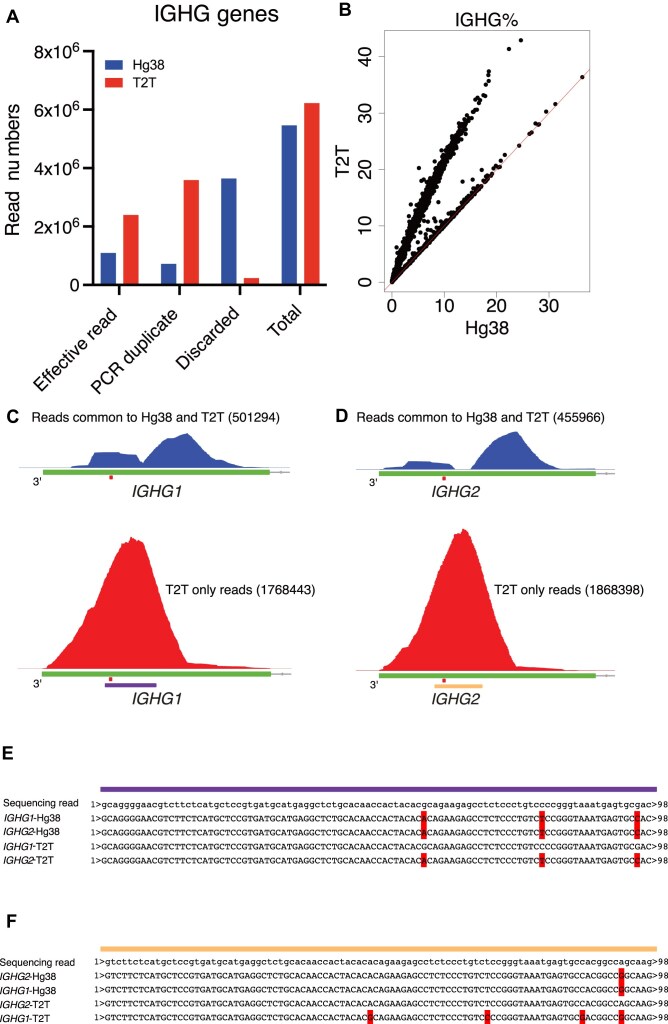
T2T reference results in more reads that are mapped to IGHG genes than hg38. (**A**) Classification of reads that are assigned to IGHG genes for the BMPC dataset for each reference genome. The bar plots show the total read count of IGHG genes and also the number of effective reads, the number of reads identified as PCR duplicates, and the number of reads discarded for other reasons. (**B**) Percentage of IGHG gene reads per cell for the BMPC dataset. hg38 and T2T results are compared by scatter plots. Red lines in scatter plots represent the *x* = *y* line. (**C**) Top: the coverage plot from Supplementary Fig. S11C with the same scale as the bottom coverage plot; bottom: the coverage plot of reads that map to IGHG1 perfectly using T2T but with ambiguity when using hg38. The small red bar denotes the location of the stop codon. The numbers of reads that are inputted to generate the indicated coverage plots are also shown for the aim of comparison. The purple bar indicates the location of the sequencing read of panel (E). (**D**) Top: the coverage plot from Fig. 3D with the same scale as the bottom track plot; bottom: the coverage plot of reads that map to IGHG2 perfectly using T2T but with ambiguity when using hg38. The small red bar denotes the location of the stop codon. The numbers of reads that are inputted to generate the indicated coverage plots are also shown for the aim of comparison. The yellow bar indicates the location of the sequencing read of panel (F). (**E**) Alignment results for one illustrative sequence read with assignment ambiguity between IGHG1 and IGHG2 when using hg38 in panel (C). The full sequence of the read is shown together with the best matches to the IGHG1-hg38, IGHG2-hg38, IGHG1-T2T, and IGHG2-T2T genome regions. Mismatched bases are marked in red. The read maps perfectly to IGHG1-T2T and, using T2T, the assignment to IGHG1 is unambiguous. Using hg38, however, the result is ambiguous with the same number of mismatches between IGHG1-hg38 and IGHG2-hg38. (**F**) Alignment results for one illustrative sequence read with assignment ambiguity between IGHG1 and IGHG2 when using hg38 in panel (D). The full sequence of the read is shown together with the best matches to the IGHG2-hg38, IGHG1-hg38, IGHG2-T2T, and IGHG1-T2T genome regions. Mismatched bases are marked in red. The read maps perfectly to IGHG2-T2T and, using T2T, the assignment to IGHG2 is unambiguous. Using hg38, however, the result is ambiguous with the same number of mismatches between IGHG2-hg38 and IGHG1-hg38.

**Figure 5.**
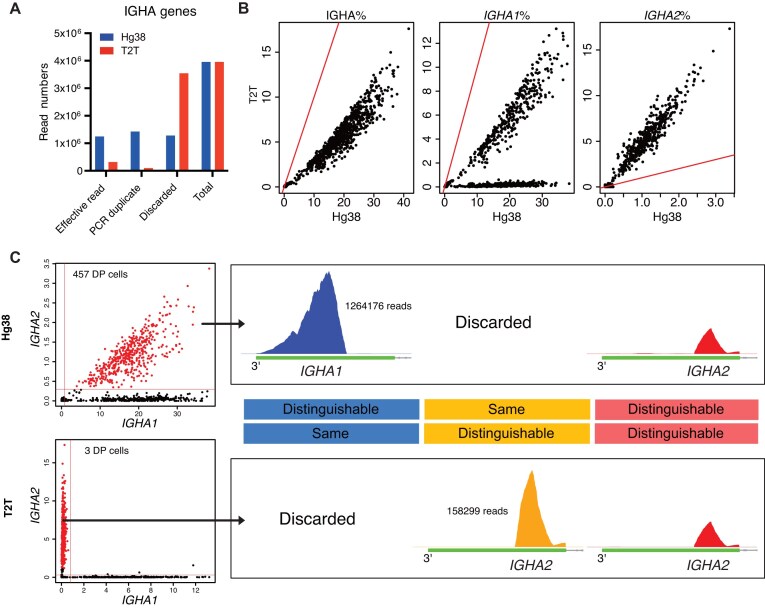
T2T output has fewer IGHA reads but better distinguishes IGHA1 from IGHA2. (**A**) Classification of reads that are assigned to IGHA genes for the intestinal mucosa (intestine) dataset for each reference genome. The bar plots show the total read count of IGHA genes and also the number of effective reads, the number of reads identified as PCR duplicates, and the number of reads discarded for other reasons. (**B**) Percentage of IGHA gene reads per cell, IGHA1 reads per cell, and IGHA2 reads per cell for the intestine dataset. hg38 and T2T results are compared by scatter plots. Red lines in scatter plots represent the *x* = *y* line. (**C**) The left scatter plots correlating expression levels of IGHA1 and IGHA2 using hg38 (top) and T2T (bottom). The IGHA1^+^ IGHA2^+^ cells of the hg38 output are highlighted in both plots. The horizontal and vertical red lines represent the thresholds for positive cells of an isotype gene. Here, the thresholds are 0.8% and 0.3% respectively, for IGHA1 and IGHA2. The numbers of DP cells for both outputs are also shown. In the right, the plots on the top show coverage plots for reads aligned using hg38 from the highlighted cells in the scatter plots. Top left plot shows reads mapped to IGHA1 using hg38 (these reads are discarded when using T2T because of mapping ambiguity) and top right shows reads mapped to IGHA2 using hg38 (these reads also map IGHA2 using T2T). Only the CH3-CHS exon of the genes is shown. The plots on the bottom show coverage plots for reads aligned using T2T. The bottom middle plot shows reads that map IGHA2 perfectly, and using T2T, the assignment to IGHA2 is unambiguous (these reads are discarded when using hg38 due to mapping ambiguity). The bottom right plot shows that all reads mapped to IGHA2 using hg38 map also map to IGHA2 using T2T. The numbers of reads that are inputted to generate the indicated coverage plots are also shown. A schematic plot of the CH3-CHS exons of IGHA1 or IGHA2 is between the hg38 and T2T coverage plots. The text explains the result of sequence alignment between IGHA1 and IGHA2 for the T2T or hg38 genomes. The colours used here have the same meaning as the coverage plots, i.e. the reads of coverage plots with one colour locate on the region in the same colour.

The matrix output from cellranger count was further processed following the general Seurat [42] pipeline, in which quality control (QC), normalization, and dimension reduction are performed. QC involved four steps: (i) cells that have >10% mitochondrial gene expression were removed; (ii) ribosomal genes were removed; (iii) genes that are expressed in <0.5% (0.25% for the intestine mucosa dataset) of total cells were removed; and (iv) cells with too few reads (usually 1500 as threshold) or expressed genes (usually 450 as threshold) were removed (Supplementary Fig. S3 and Supplementary Table S1). Finally, only cells that have >10% immunoglobulin gene expression (Supplementary Fig. S4) and appear in both hg38 and T2T outputs are defined as ASCs for downstream analysis and used for generating Figs 1B, 2, 3A, 4B, and 5B and C (scatter plots) together with Supplementary Figs S5, S6, S7, S8, S9, S11A, S12B, S14B, S15B, S16B, S17B, and S18 scatter plots.

**Figure 6.**
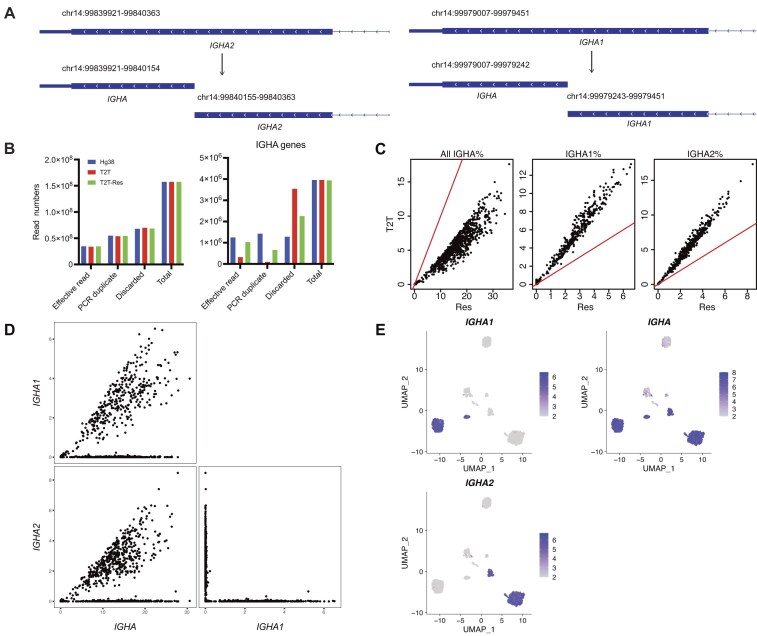
A custom annotation reference for T2T can rescue multi-mapped reads mapped to IGHA genes. (**A**) Schematic plots showing the CH3-CHS exons of IGHA2 and IGHA1, and how they are split into two exons, respectively. Coordinates of the exons are shown in the top of each exon. Gene names of the exons are shown in the bottom of each exon. Within each exon, the arrows denote the direction of the gene, the thicker blue bar indicate the protein coding region, and the thinner blue bar indicate the 3-UTR region. (**B**) Classification of reads that are of the whole dataset (left) or are assigned to IGHA genes (right) for the intestinal mucosa datasets for each reference. The bar plots show the total read count and also the number of effective reads, the number of reads identified as PCR duplicates, and the number of reads discarded for other reasons. Only effective reads were used for downstream analyses. (**C**) Percentage of IGHA gene reads (left), IGHA1 reads (middle), and IGHA2 reads (right) per cell for the intestine dataset. hg38 and T2T results are compared by scatter plots. Red lines in scatter plots represent the *x* = *y* line. T2T-res is shown as ‘res’ in plots. (**D**) Paired scatter plots correlating the expression levels of pairs of IgA subclass genes for intestinal mucosa ASCs, using the custom reference based on T2T. Expression is represented as reads per hundred (RPH). (**E**) UMAP dimension reduction plots of individual ASCs for the intestinal mucosa dataset, using the custom output based on T2T. Cells are coloured by expression (RPH) of the indicated gene for a particular IgA subclass.

**Figure 7.**
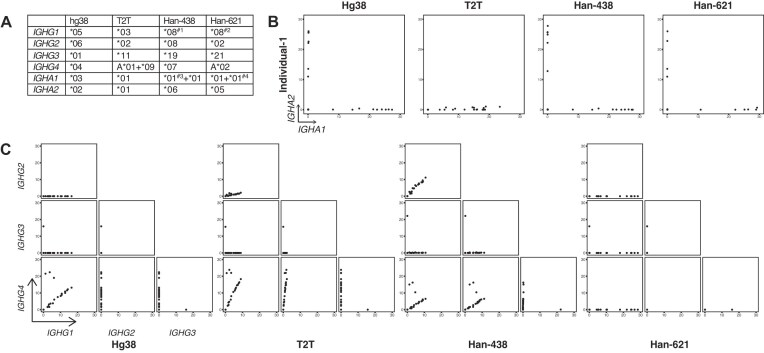
The allele differences between genome references and their impact on isotype calling. (**A**) The table shows the IMGT alleles of isotype genes in four genome references: #1, there is one mismatch in the protein coding region and three in the 3-UTR; #2, there are five mismatches in the 3-UTR; #3, IGHA1 is duplicated in Han-438, and for the first IGHA1, there is one mismatch; and #4, IGHA1 is duplicated in Han-621, and for the second IGHA1, there is one mismatch. (**B**) Scatter plots correlating the expression levels of IGHA1 and IGHA2 for intestinal mucosa ASCs of the first Chinese individual. The genome references used for each plot are shown accordingly. Here, expression is represented as RPH. (**C**) Paired scatter plots correlating the expression levels of pairs of IgG subclass genes for intestinal mucosa ASCs of the first Chinese individual. The genome references used for each plot are shown accordingly. Here, expression is represented as RPH.

To generate Figs 6C–E and 7B and C together with Supplementary Figs S20B and C, and S21, the similar process was performed but with the custom references.

### Alignment track plot visualization

To generate Figs 3C, 4C and D, and 5C, and Supplementary Fig. S11B, BAM files of particular genes and cells were generated by samtools based on the read names, CR tag (representing cell barcodes), GN tag, and xf tag (only effective reads and PCR duplicate reads were included). Alignment track plots were produced from the BAM files with the R package Gviz [43].

### Sequence alignment

To determine the allotype alleles, an online tool, IMGT/BlastSearch [44], was used based on the databases of IMGT [45, 46].

A plasmid Editor (ApE) software [47] (https://jorgensen.biology.utah.edu/wayned/ape) was used to generate Figs 3D and 4E and F, and Supplementary Figs S11C, S13, S14A, S15A, S16A, S17A, and S19.

## Results

### Mapping performance is comparable between hg38 and T2T

To compare the performance of hg38 and T2T in isotype identification, two human 10x Genomics Chromium 3′ scRNA-seq datasets were selected, one generated in-house from enriched BMPCs from one individual [33] and the other from intestinal mucosae of one individual published in [34]. Two supplementary 10x Genomics Chromium 3′ scRNA-seq datasets from [35] (one tonsil samples) and [36] (one bone marrow sample, BM) were also interrogated to demonstrate the reproducibility of the results. Equivalent custom Cell Ranger references were constructed from the hg38 and T2T genomes, and then cell-by-gene matrices of read counts were obtained from Cell Ranger. Examining the alignment output BAM files, mapping performances of hg38 and T2T at the read level were similar, with approximately the same numbers of effectively mapped sequence reads and PCR duplicates (Fig. 1A and Supplementary Fig. S2A). The edit distances and mapping scores of the reads were also comparable (Supplementary Fig. S2B). After the read alignment and cell demultiplexing, QC was performed on all single cells (Supplementary Table S1 and Supplementary Fig. S3). The QC metrics were similar between the hg38 and T2T output (Supplementary Fig. S3). These results show that there are no global differences between the hg38 and T2T genomes, and that the two genomes have been processed in equivalent ways in our pipeline. After QC, *bona fide* ASCs were selected based on immunoglobulin gene expression and only cells appearing in both hg38 and T2T outputs were retained (Supplementary Fig. S4). Read and gene coverage per ASC cell was also comparable between hg38 and T2T (Fig. 1B and Supplementary Fig. S5). In this study, ASCs are the target cells for isotype detection. Expression levels of two key marker genes, JCHAIN [48] and XBP1 [49], were similar between the hg38 and T2T outputs in all datasets (Fig. 1B and Supplementary Fig. S5B). Taken together, the general qualities of single ASCs have few differences between the hg38 and T2T reference genomes.

### T2T but not hg38 identifies immunoglobulin isotypes correctly

We next examined expression levels of the immunoglobin heavy chain (IGH) genes that are associated with isotype switching of ASCs. Plotting the expression of IGHA, IGHD, IGHE, IGHG, and IGHM in the ASCs did not reveal any unexpected patterns (Supplementary Fig. S6). Almost every ASC can be seen to express exactly one of these variants, and the small number of exceptions can be interpreted as likely doublet cells (Supplementary Fig. S6). The pattern was similar for both hg38 and T2T. The proportion of reads assigned to IGHM, IGHD, and IGHE for each ASC correlated closely between hg38 and T2T (Supplementary Fig. S7).

Drilling down to the IGH subclass genes showed a different story, however. UMAP plots of ASC cells in the BMPC dataset, overlaid with IGHG1, IGHG2, IGHG3, and IGHG4 expression, showed that most cells expressed exactly one isotype gene when T2T was the reference genome, but many cells unexpectedly expressed two isotypes simultaneously with hg38 as the reference (Fig. 2A). A scatterplot of IGHG1 versus IGHG2 shows the problem very clearly (Fig. 3A). With T2T as the reference, a handful of doublet cells can be seen, but almost all cells express exactly one isotype and are positioned on the vertical or horizontal axes. With hg38 as the reference, a large number of cells (759 cells) are positioned on a non-vertical trend line and apparently express both IGHG1 and IGHG2 (Fig. 3A, left). The DP cells cannot be interpreted as doublet cells because the frequency is so much greater than that observed for doublet cells in Supplementary Fig. S6, and because doublet cells would be randomly positioned rather than systematically arranged on a trend line. We can confirm this interpretation by examining the total reads per cell (library sizes) of the DP cells. The T2T DP cells have nearly twice the reads per cell on average compared to non-DP cells (*P*= .000022), confirming they are genuine doublets, whereas the uniquely hg38 DP cells are similar to non-DP cells in terms of reads per cell (Fig. 3B). The same problem occurs with DP cells for IGHG1 and IGHG3 (Supplementary Figs S8 and S11A). The hg38 output generates 1141 IGHG1^+^IGHG3^+^ DP cells, on a broad diagonal trend line, whereas the T2T output shows only single-positive cells apart from a very small number of apparent doublets (Supplementary Fig. S11A).

A similar situation occurs for the IgA isotype genes, IGHA1 and IGHA2. Plotting IGHA1 versus IGHA2 expression for ASCs in the intestine mucosa dataset showed that half the cells were DP for hg38 but almost none for T2T (Figs 2B and 5C). The same pattern is seen for all the datasets (Supplementary Fig. S9).

The high rate of DP cells in the hg38 output is not compatible with the ‘one-cell-one-antibody’ theory, and indicates incorrect alignment of sequence reads to the hg38 genome.

### DP cells in the hg38 output should be single-positive

To explore why the T2T genome performs better for isotype identification, we focused first on IGHG1^+^IGHG2^+^ DP cells and examined track graphics for the corresponding read alignments. The cells highlighted in red in Fig. 3A show high expression of both IGHG1 and IGHG2 in the hg38 output but express only IGHG2 in the T2T output. To understand why this occurs, we examined the alignment positions of all the relevant reads in the hg38 output. Due to the Chromium 3′ sequencing, most of the reads from immunoglobulin transcripts were mapped to constant regions encoding the isotype genes (Supplementary Fig. S1). Reads were in particular mapped to the most 3′ exon of the secreted isoforms, which for IGH is the CH3-CHS exon (constant heavy chain exon 3). The left panel of Fig. 3C shows read alignment coverage plots for the CH3-CHS exons of IGHG1 and IGHG2 in the hg38 output. The plots show all reads from the ‘red’ cells in Fig. 3A that map to either IGHG1 or IGHG2 in the hg38 output. The reads mapping to IGHG1 are responsible for IGHG1 expression in cells that are primarily IGHG2 in the hg38 output.

The right panel of Fig. 3C shows the T2T coverage for the same reads as in the left panel. When aligned to T2T, the reads in the main IGHG1 peak in the hg38 output were remapped to the homologous region of IGHG2 instead of to IGHG1. The edit distances and mapping scores of these reads showed fewer mismatches and higher mapping quality when realigned to T2T instead of hg38 (Supplementary Fig. S10).

Comparing the consensus sequence of reads around the 3′ end region with the sequence of the 3′ end regions of IGHG1 and IGHG2 from both hg38 and T2T, the numbers of mismatches (red bases) were 2, 1, 0, and 7 for IGHG2-hg38, IGHG1-hg38, IGHG2-T2T, and IGHG1-T2T, respectively (Fig. 3D). Within hg38 output, IGHG1 had fewer mismatches than IGHG2; thus, those reads were mapped to IGHG1 causing the DP cells. However, in T2T output, it was the IGHG2 that had zero mismatch; hence, reads were mapped to IGHG2 (Fig. 3D). Taking these together, the IGHG1^+^IGHG2^+^ cells in the hg38 output should be IGHG2 single-positive cells, because the reads in the 3′ end region from IGHG2 single-positive cells in the real world are mapped to IGHG1 incorrectly when using hg38 as genome reference.

Using a similar checking process, it is apparent that the IGHG1^+^IGHG3^+^ DP cells, in the hg38 output, should be IGHG1 single-positive cells (Supplementary Fig. S11).

### The T2T reference results in more IGHG reads than hg38

In addition to higher accuracy in isotype identification, the T2T output also has more reads that are mapped to IGHG genes. Checking the BAM files, there were more effective reads and PCR duplicates mapped to IGHG genes in the T2T output than the hg38 output because many reads were discarded in the hg38 output (Fig. 4A and Supplementary Fig. S12A). At single-cell level, many cells in the T2T output gained more IGHG gene expression than hg38 (Fig. 4B and Supplementary Fig. S12B). Rechecking the IGHG1^+^ (red cells) of T2T output indicated in Supplementary Fig. S11A, in addition to 501 294 reads that were also mapped to IGHG1 or IGHG3 in the hg38 output (Supplementary Fig. S11B, the right two track plots), 1 768 443 extra reads around the IGHG1 stop codon were recovered (Fig. 4C). Similarly, for the IGHG2^+^ (red cells) of T2T output indicated in Fig. 3, in addition to 455 966 reads that were also mapped to IGHG2 or IGHG1 in hg38 output (Fig. 3C, the right two track plots), 1 868 398 extra reads around IGHG2 stop codon region were recovered (Fig. 4D). Tracking those extra recovered reads revealed that they were discarded in the hg38 output because they mapped to homologous regions of IGHG1 and IGHG2 and, hence, were discarded by the Cell Ranger software as multi-mapping reads with no unique best location. In contrast, the sequences of T2T, at the same locations, were distinguishable between IGHG1 and IGHG2, so reads could be mapped to IGHG1 (Fig. 4E) or IGHG2 (Fig. 4F) without ambiguity.

To further understand the discrepancies of IGHG genes between T2T and hg38, the sequences of the CH3-CHS exons of IGHG3, IGHG1, IGHG2, and IGHG4 from both T2T and hg38 were compared. There were in total 34 variants in the CH3-CHS exons among four IGHG genes in T2T (Supplementary Fig. S13A) but only 28 variants in hg38 (Supplementary Fig. S13B), causing the T2T sequences to be more distinguishable than those for hg38.

For IGHG3, the relevant sequences of IGHG3 in T2T and hg38 were identical except for one variant near the end of the 3-UTR (Supplementary Fig. S14A) and, as a result, the expression levels of IGHG3 in *bona fide* IGHG3^+^ cells (the dots around the *x* = *y* red line in Supplementary Fig. S14B) were comparable between T2T and hg38 (Supplementary Fig. S14B). The dots far from the *x* = *y* lines in Supplementary Fig. S14B represent IGHG1^+^ cells, some of whose reads were assigned to IGHG3-hg38.

For IGHG1, the sequences were different between T2T and hg38 for both protein-coding regions (five mismatches) and 3-UTRs (eight mismatches) (Supplementary Fig. S15A). As a result, the alignments and gene assignments of reads changed as shown in Fig. 3D (IGHG1-hg38 to IGHG2-T2T), Supplementary Fig. S11C (IGHG3-hg38 to IGHG1-T2T), and Fig. 4E (extra reads for IGHG1-T2T), and the consequent expression levels were also altered between T2T and hg38, T2T output having higher expression in IGHG1^+^ cells (Supplementary Fig. S15B).

For IGHG2, there was only one variant in the protein-coding regions but three variants in the 3-UTRs (Supplementary Fig. S16A). The number of mismatches between hg38 and T2T is fewer than for IGHG1, but still resulted in higher IGHG2 expression in IGHG2 single-positive cells in the T2T output (Supplementary Fig. S16B).

For IGHG4, T2T has two copies, which are found in 44% of humans [50]. Copy 1 (the more 5′ upstream of the two) had two variants in the protein-coding region compared with hg38 but shared the same 3-UTR sequence (Supplementary Fig. S17A). Copy 2 had a nearly identical protein-coding region as hg38, with just one variant, but had a distinct 3-UTR containing six variants (Supplementary Fig. S17A). Thus, most of reads that could be mapped to IGHG4 of hg38 could also be assigned to either one of the IGHG4 genes of T2T, which was proved by the comparable expression levels of IGHG4 between T2T and hg38 (Supplementary Fig. S17B).

To conclude, the sequence differences of IGHG1 and IGHG2 between hg38 and T2T brought about fewer multi-mapping reads and higher IGHG gene expression in the T2T output.

### T2T yields fewer IGHA reads but better distinguishes IGHA1 and IGHA2

Unlike IGHG, there were fewer reads mapped to IGHA genes in the T2T output compared with hg38 [Fig. 5A and B (left), and Supplementary Fig. S18 (left two columns)]. This read reduction was associated with lower IGHA1 expression [Fig. 5B (middle) and Supplementary Fig. S18 (third column)] but higher IGHA2 expression [Fig. 5B (right) and Supplementary Fig. S18 (fourth column)].

To understand this phenomenon in detail, we aligned the sequences of the CH3-CHS exons of IGHA1 and IGHA2 in the hg38 and T2T genomes, and divided them into three regions, marked as ‘blue’, ‘orange’, and ‘red’ respectively, in Fig. 5C and Supplementary Fig. S19.

In the ‘blue’ region, the IGHA1 and IGHA2 sequences are identical in the T2T genome but slightly different in hg38. Reads mapping to this region were therefore discarded in the T2T output, because of multi-mapping, but mapped to IGHA1 in the hg38 output (Fig. 5C). The ‘blue’ region includes the 3-UTRs of the two genes, together with the 3′ tail of the coding regions where there are nine variants between IGHA1 and IGHA2 in hg38 but none in T2T (Supplementary Fig. S19).

In the ‘orange’ region, the IGHA1 and IGHA2 sequences are identical in the hg38 genome but distinguishable in T2T, so reads mapping this region are discarded in the hg38 output but mapped to IGHA2 in the T2T output [Fig. 5B (right) and C]. There is one variant distinguishing IGHA1 from IGHA2 in T2T (Supplementary Fig. S19), defining the orange region (Fig. 5C and Supplementary Fig. S19).

Sequences in the ‘red’ region are distinguishable in both hg38 and T2T, so reads mapping to this region were mapped to the appropriate genes correctly in both outputs (Fig. 5C).

Taking these observations together, we can see that true IGHA2 cells have become false IGHA1^+^IGHA2^+^ DP cells in the hg38 output, because reads from those cells mapping to the ‘blue’ region were incorrectly assigned to IGHA1. The same reads were discarded in the T2T output, resulting in lower IGHA1 expression and avoidance of false DP cells. Meanwhile, T2T sequences are distinguishable in the ‘orange’ region, resulting in higher IGHA2 expression.

### A custom annotation reference for T2T can rescue multi-mapped reads mapped to IGHA genes

To quantify IGHA gene expression more precisely and rescue reads mapped to the ‘blue’ region (Fig. 5C), a custom annotation reference for T2T was created, in which the CH3-CHS exons of IGHA2 and IGHA1 were split into two ‘exons’, respectively (Fig. 6A). The 3′ most of the newly constructed exons, sharing the identical sequences (Supplementary Fig. S19), were named as IGHA, a new artificial gene, and were approximately equivalent to the ‘blue region’ (Supplementary Fig. S19). When reads were mapped to IGHA exons, they would not be discarded even though they were multi-mapped reads because Cell Ranger retrieved multi-mapped reads if the regions they were mapped to pointed to the same gene, in this case IGHA. After this custom editing, effective reads assigned to IGHA genes were largely increased without influencing the general effective read numbers (Fig. 6B and Supplementary Fig. S20A and B). At the single-cell level, the expression of general IGHA genes also increased [Fig 6C (left) and Supplementary Fig S20B], but the expression of both IGHA1 and IGHA2 decreased [Fig. 6C (middle and right) and Supplementary Fig. S20B], implying that the increase in general expression was attributed to the new IGHA gene. Looking at the scatter plots of the expression levels of IGHA1, IGHA2, and IGHA, most of the IGHA1^+^ or IGHA2^+^ single cells are also IGHA positive, indicating that those single-positives had gained the rescued reads from the IGHA exons (Fig. 6D and Supplementary Fig. S20C). Inheriting the advantages of T2T, this custom reference did not produce IGHA1 and IGHA2 DP cells (Fig. 6D and Supplementary Fig. S20C) and had a well-separated clustering based on the isotype expression (Fig. 6E). Therefore, the custom annotation reference could effectively overcome the issue of lost IGHA gene reads.

### The impact of allele differences on isotype calling

The above results demonstrate that sequence discrepancies caused different alignment outcomes between hg38 and T2T for reads mapped to isotype genes. To determine the origins of those sequence variants, the full sequences including protein-coding regions and 3-UTRs of each IGHG and IGHA gene from both hg38 and T2T were aligned to the database of IMGT (https://www.imgt.org/blast/). The alignments indicated that hg38 and T2T represented different alleles for each IGHG and IGHA gene (Fig. 7A). Bashirova *et al.* [51] performed a comprehensive analysis of the allele distributions of IGHG1, IGHG2, and IGHG3 between different ancestries, and showed that the most common haplotype of these three genes for European American was IGHG3*11/IGHG1*03/IGHG2*02, the same haplotype as T2T, while the hg38 haplotype IGHG3*01/IGHG1*02/IGHG2*06 was common in sub-Saharan African, matching the local ancestries of the each genome [6]. The allele population distributions of IGHG4, IGHA1, and IGHA2 remained to be investigated.

The genetic background of the in-house BMPC dataset is European Australian. While such information was lacking for the other three non-Chinese public datasets, it is reasonable to assume that they are also of European origin, based on the similar isotype calling outcomes and the fact that the dataset samples were collected in the USA and in Britain. Thus, it was reasonable that the European-origin datasets yielded better results when using T2T as the genome reference. Conversely, mapping the European-origin datasets to the African-origin hg38 gave rise to false results that did not obey the ‘one-cell-one-antibody’ dogma.

In [51], no haplotype analysis was performed for an East Asia population, but they did show that the most frequent alleles of each single gene for East Asia were different from the European or African alleles. To investigate whether T2T also performs better for East Asian data, an additional 10x Genomics Chromium single-cell 3′ dataset including two Han Chinese individuals was examined [37]. The 10x data for the two Chinese individuals were mapped to hg38 and T2T, and also to two Han Chinese Han genomes from [40]. Han-438 and Han-621 display alternative alleles compared with hg38 and T2T (Fig. 7A). With the same QC and downstream analysis process, the isotype calling was visualized by scatter plots (Fig. 7B and C, and Supplementary Fig. S21).

For IGHA genes, with the ‘one-cell-one-antibody’ as a ground truth, hg38, Han-438, and Han-621 all displayed good IGHA isotype calling (Fig. 7B and Supplementary Fig. S21B). In contrast, the T2T output of individual 1 only displayed IGHA1^+^ cells, missing IGHA2 expression, and the T2T output of individual 2 displayed IGHA DP cells.

For IGHG genes, hg38, T2T, and Han-438 did not provide accurate immunoglobulin isotype calling, with all displaying IGHG DP cells (Fig. 7C and Supplementary Fig. S21C). Only Han-621 provided a relatively better output.

It remains challenging to interrogate the isotypes for East Asians due to the rareness of public datasets including ASCs highly expressing isotype genes and due to the lack of a complete and representative East Asian genome assembly. Based on the preliminary analysis of these two Chinese individuals, it can be deduced that T2T is not always the optimal reference genome depending on the ancestry of the subjects.

## Discussion

While model organisms, such as laboratory mice, have very uniform genetic backgrounds, human individuals have very diverse genetic backgrounds. Despite this known sequence diversity, hg38 was the only human genome reference in standard use until the relatively recent publication of T2T. Whether the variable human genetic backgrounds impact the outcome of sequencing data analyses with a single genome reference remains largely unexplored. Here, our analysis indicated that two genome references, hg38 and T2T, with different local ancestries, provided different results for IGH isotype calling with single ASC RNA-seq data.

‘One-cell-one-antibody’ is a cornerstone of the clonal selection theory that has been central to our understanding of the immune system for over 60 years [52]. This theory can be employed as a ground truth, where one single cell should only express one isotype gene. When analysing the four public datasets, the T2T output fitted this theory, while the widely used hg38 output did not, prompting us to explore the underlying cause of this uncertainty.

The mapping performances of hg38 and T2T were similar on a genome-wide level, with the notable exception of the immunoglobulin isotype genes. The hg38 outputs displayed many IGHG1^+^IGHG2^+^, IGHG1^+^IGHG3^+^, and IGHA1^+^IGHA2^+^ isotype DP cells. It was unlikely that the dual expression was the result of cell doublets being sequenced together, as these events should be randomly distributed between any two isotypes, instead of just between certain isotypes, as observed. In the T2T outputs, the cells were all single-positive, apart from a negligible number of doublet cells. The identity of DP cells as doublets in the T2T output but not in the hg38 output was confirmed by checking the average number of reads per cell. The number of detected genes per cell showed the same trend (data not shown). The cells that were IGHG1^+^IGHG2^+^ in the hg38 output became IGHG2^+^, cells that were IGHG1^+^IGHG3^+^ became IGHG1^+^, and cells that were IGHA1^+^IGHA2^+^ became IGHA2^+^. The conversions were due to the correct assignments of reads that were mapped to certain regions of the 3′ exons (Figs 3D and 5C, and Supplementary Fig. S11C).

In addition, because the sequences of IGHG gene family are more distinguishable in T2T than hg38, many reads that were multi-mapped in hg38 could be assigned to IGHG1 and IGHG2 in the T2T outputs (Fig. 4). However, the situation for IGHA genes was reversed. Due to a large segment of homologous sequences between IGHA1 and IGHA2 in T2T, reads mapped to this segment were regarded as multi-mapping reads and were discarded in the T2T output (Fig. 5F). We showed that those discarded reads could be rescued with a modified reference (Fig. 6). Our use of a modified genome here is analogous to the use of modified genomes to deal with erroneous sequences [53] or to recover reads from highly identical paralogs [54], although we are the first to apply this technique to immunoglobin isotypes.

Next, we elaborated why the isotype sequences were different between these two genome references. T2T is the first published complete human genome to employ long-read sequencing technology [5]. T2T is sequenced from a homozygous complete hydatidiform mole (CHM) cell line, representing a complete haplotype from a single European individual [5]. In comparison, the current hg38 genome is derived from multiple individuals, with one individual with African–European admixed ancestry dominating [4]. The alleles of IGHG1, IGHG2, and IGHG3 of T2T and hg38 are distinct and represent the most frequent haplotypes of European and sub-Saharan African ancestry, respectively [51]. Thus, it is reasonable that the isotype sequence differences are reflected by the ancestral discrepancy. The ancestry of the BMPC data is known to us and is of European origin. For the other three non-Chinese public datasets, ancestral information was not released, but it seems likely that they are also European origin because they have similar isotype calling results to the BMPC data. It could be speculated that mapping European samples to T2T results in better outcomes than mapping to hg38, which reflects a different ancestral genetic background. To address the impact of the genetic background of the genome reference, we introduced one more public dataset from China and demonstrated that neither the European-based T2T nor the African-based hg38 performed better for Chinese samples than the native Han genome references.

Considering the importance of immunoglobulin isotypes in immune regulation and diseases, and also in the monoclonal antibody production industry, it is necessary to identify the isotypes correctly. Based on this study, when using the mapping count method with a single genome reference, T2T genome reference is a better option than hg38 for European-origin sample but not for East Asian samples. In each circumstance, the genetic background of the genome reference will impact the results of the sequencing data analysis, at least for the isotype calling, but perhaps for other gene families as well that are highly variable between human populations.

The aim of the T2T genome project was to fill in gaps that were present in previous drafts of the human genome, particularly in complex regions such as centromeres and telomeres. The T2T also provides a genome reference with European genetic background. Our study shows that the improvements offered by the T2T genome are not limited to the newly completed regions but, for European samples, are also present in terms of improved accuracy for a protein-coding gene family that was fully covered in older genomes. In the future, it may be possible to use pangenome references to improve read alignment results across a wide range of human subpopulations simultaneously, although this is not yet practical for routine scRNA-seq analysis [40, 55].

Our study was limited to immunoglobin genes because it is only for that family that the clonal selection hypothesis of ‘one-cell-one-antibody’ allows us to identify and diagnose read alignment inaccuracies. There is no reason to expect that reference bias should be limited to this one gene family, but the isotype results are especially sensitive to such biases because of the high level of sequence similarity between the isotype subclass genes. Our study used 10x Genomics Chromium data with 3′ short-read sequencing, because this technology is currently the most popular platform for routine scRNA-seq profiling. Emerging technologies offer the possibility of long-read single-cell RNA sequencing, which might ameliorate the impact of reference biases for isotype identification and expression profiling, but this remains to be explored [56].

## Supplementary Material

lqaf074_Supplemental_File

## Data Availability

The datasets analysed during the current study are available from the NCBI Sequence Read Archive experiments: https://www.ncbi.nlm.nih.gov/sra/SRX20001597, https://www.ncbi.nlm.nih.gov/sra/SRX6584269, https://www.ncbi.nlm.nih.gov/sra/SRX9986858, and https://www.ncbi.nlm.nih.gov/sra/SRX4720060. Computer code used to generate results in this manuscript is provided in the Supplementary material.
